# A Theoretical Model for Urban Walking Among People With Disabilities

**DOI:** 10.3389/fpsyg.2020.00156

**Published:** 2020-02-13

**Authors:** Elizabeth Marcheschi, Agneta Ståhl, Mai Almén, Maria Johansson

**Affiliations:** ^1^Chalmers University of Technology, Gothenburg, Sweden; ^2^Swedish University of Agricultural Sciences, Alnarp, Sweden; ^3^Transport and Roads, Department of Technology and Society, Lund University, Lund, Sweden; ^4^HinderfriDesign AB, Lund, Sweden; ^5^Environmental Psychology, Department of Architecture and Built Environment, Lund University, Lund, Sweden

**Keywords:** urban walking, people with disabilities, theoretical model, environmental psychology, traffic planning

## Abstract

This paper is an attempt to advance research on walking at a neighborhood level of analysis for people with disabilities by proposing a theoretical model that combines the knowledge of two disciplines: traffic planning and environmental psychology. The aim is to provide guidance for a discussion and a plan for future interdisciplinary investigations by proposing a model that accounts for the dynamic interaction between environmental characteristics, human processes, and walking experience among individuals with a disability. For this purpose, traffic planners, and environmental psychologists came together to discuss theories, concepts, and thematic relevance in a series of focus group meetings. These meetings led to the selection of the Human Environment Interaction (HEI) model, originally developed from the field of environmental psychology and operationalized to describe how walking experiences result from the interplay between individual abilities, emotional processes, and the physical and social characteristics of the environment (Küller, 1991). The proposed model aims to sustain interdisciplinary discussion and research planning around the topic of neighborhood walking for people with disabilities. By operationalizing each dimension in the model, a good fit between groups with disabilities and individual differences associated with walking experiences is assumed, which, in turn, will have the potential to provide a more conscious analysis of wellbeing-related outcomes, such as usability of the environment, frequency of mobility, and quality of life. However, to improve understanding of urban walking at a neighborhood level for people with disabilities, empirical studies must be carried out to test the proposed model.

## Introduction

Sustainable development goals call for better opportunities for everyone to walk, cycle, and travel by public transport in urban areas (UN Agenda 2030, SDG 11). Walking has been recognized as one of the most affordable and easy behaviors that a human can perform to support health outcomes (Kerr et al., 2012). Furthermore, walking fosters community participation and environmental sustainability, promoting thereby a new form of urbanism and environmental growth that reduces greenhouse effects (Saelens et al., 2003; Lee and Buchner, 2008; Orru and Orru, 2010). As a consequence, a considerable amount of research, policy measures, and guidelines on how to design human-friendly walkable communities have been produced (Middleton, 2010; Van Holle et al., 2012; Harris et al., 2013). Also, the neighborhood has received special attention since this is where people interact regularly and most walking activities occur (Amérigo, 2002; Brown et al., 2007; Shigematsu et al., 2009; Bonaiuto and Alves, 2012; Van Dyck et al., 2013; Ferreira et al., 2016; Johansson et al., 2016). However, these research efforts are primarily based upon a view of pedestrians that does not include disabilities, failing, therefore, to capture human diversity and needs across vulnerable groups of society such as that of people with different types of functional abilities (World Health Organization [WHO], 2011; Stafford and Baldwin, 2018).

Historically, research theories and methodologies addressing walking behaviors have largely focused upon identification of urban features, environmental design qualities, and transport system characteristics that encourage walking activities among the general population (Owen et al., 2004; Ewing and Handy, 2009; Ewing and Cervero, 2010). As an example, the public health paradigm has primarily focused upon the incidental health benefits associated with walking, whereas the traffic planning paradigm has shed light on environmental design features that encourage walking among a healthy population (Owen et al., 2004, 2011; Sallis et al., 2008; Wennberg et al., 2009; Ståhl et al., 2013; Hallgrimsdottir and Ståhl, 2016). On the other hand, people with disabilities, such as mobility-, vision-, hearing-, cognitive-, and hypersensitivity-related impairments, have been overlooked (Ståhl et al., 2008, 2010, 2013; Wennberg et al., 2009). In turn, this has generated a simplified understanding of which urban features are supportive for walking, and this results in a standardization of guidelines that prolongs social and mobility exclusion of certain groups in society (Andrews et al., 2012). However, recent discussions have also put forward the importance of developing a more sustainable, safe, and inclusive urban environment through consideration of how urban planning, design, and maintenance may facilitate walking activities of people with disabilities.

In this regard, Stafford and Baldwin (2015; 2018) point to the need to re-think the lack of theoretical models and interdisciplinary investigations that account for the variation of walking needs across different vulnerable groups. Therefore, a further understanding of human diversity as a basis for incorporating universal design solutions for walking is necessary to counteract spatial and social marginalization of vulnerable groups of society (Stafford and Baldwin, 2018).

Today, a greater awareness about the complexity of the human-environment interaction involved in walking activities is needed (i.e., dynamic interaction of micro- and macro-environmental factors, as well as social and psychological processes), particularly when discussing people with disabilities (Metz, 2000; Forsyth and Southworth, 2008; Kirchner et al., 2008; Spinney et al., 2009; Ståhl et al., 2010; Webber et al., 2010; Carlson et al., 2012).

This is because proper function and performance of an apparently simple activity like that of walking can be a challenge (Lawton, 2001) – a challenge that, if not addressed properly, fosters spatial and social exclusion (Gleeson, 2001). The overarching aim of this study is to advance research on walking at a neighborhood level among people with disabilities by proposing a theoretical model that combines the different, yet complementary, perspectives of traffic planning and environmental psychology.

Traffic planning and environmental psychology are two disciplines that have independently considered walking behavior, with each having a special interest in the design of the physical environment for people with disabilities. Traditionally, traffic planning research has addressed the relationship between the characteristics of the physical setting (e.g., surface conditions and traffic intersections) and the actual performance of walking (physical activity), while environmental psychology has focused more on the individual experience of walking (i.e., the underlying psychological and social processes involved) and the related wellbeing outcomes (Alfonzo, 2005; Brown et al., 2007; Ferreira et al., 2016; Johansson et al., 2016; Rahm and Johansson, 2018).

The aim of this paper is to provide a theoretical model that merges the perspectives of traffic planning and environmental psychology.

The proposed model provides guidance for the development of interdisciplinary research in which the synthesis of different disciplinary knowledge supports the identification of affordances for walking activities among people with disabilities whilst also accounting for the complexity of the interactions between environmental characteristics and human processes (Huutoniemi et al., 2010). Since it is clear that policymakers and urban planners are putting great effort into the development of infrastructure for sustainable mobility (i.e., biking and walking), the proposed model is specifically intended as a starting point for interdisciplinary discourse and knowledge exchange (Banister, 2008). This aims to support the discourse among different actors surrounding sustainable modes of transport by means of a common theoretical background that can facilitate decision processes about the design of applied research investigations and the selection of methodologies to collect information about urban walking and people with disabilities.

The proposed model is applicable for investigations targeting walking experiences of people with diverse types of disabilities within urban settings (e.g., design and maintenance on a neighborhood level, such as local streets).

### Traffic Planning

Traffic planning research studies a multitude of aspects such as road users, different modes of transport, and infrastructure – anything from planning and design to maintenance issues. Traffic planning works in close collaboration with public transport and municipal authorities, discussing issues such as public transport networks, safety, and sustainable transport systems.

Transport planning, within urban settings, has traditionally focused on travel as a demand rather than an activity and on minimization of costs and environmental impact (i.e., environmentally sustainable issues). However, when it comes to walking, which perhaps is to be considered the most sustainable mode of transport both for the individual and the environment, there is a greater value to be considered than simply reaching the final destination in the most effective and efficient way (e.g., reduced time, cost, and environmental impact). For example, Banister (2008) suggested that, to promote more sustainable behaviors (e.g., walking and biking), there is a need to involve people in order to raise public acceptance toward sustainable modes of transport. Banister’s sustainable mobility paradigm (2008) was focused on the individuals’ own perception about the implications that a more active engagement in sustainable modes of transport would have on their quality of life outcomes.

This type of user-centered approach to sustainable mobility, and specifically to walking, highlights how people’s experiences are considered crucial, which appears in line with a more recent framework defined as the pedestrian decision-making process (Mateo-Babiano, 2016). This latter framework suggested that several factors are involved when people choose to walk, such as socio-demographic characteristics, purpose, frequency of travel, and attributes of the external environment. Furthermore, Mateo-Babiano (2016) suggested that, in order to develop positive walking environments, the factors mentioned above were not sufficient since there is, behind any decision to walk, a personal motivation and ability stemming from the individual’s aspirations and values associated with walking.

The centrality of the individual’s own experiences, perceptions, and needs with regard to mobility and specifically walkability has been repetitively addressed in traffic planning research using different frameworks, models, and empirical work.

However, when it comes to considering people with disabilities and their walking experience, as highlighted in this work, this has traditionally been of lower priority in the field. The research has mainly been focused on older people and people with visual impairments, and walking has often been discussed in terms of *mobility* and *accessibility in relation to the needs of these two groups* (Jenness and Singer, 2006; Ståhl et al., 2010, 2013).

Mobility is here intended as an umbrella concept that comprises different types of travel modes and refers to the ability to physically move within a community (e.g., from the home to the neighborhood and regions beyond, extending social and recreational activities as desired) (Rosenbloom, 2003). Accessibility is defined by Iwarsson and Ståhl (2003) as the relationship between the individual’s capacity and environmental demand. The definition is based on the ecological model and the environmental docility hypothesis (Lawton and Nahemow, 1973), whereas accessibility must be analyzed by an integration of information regarding both the individual and the environmental components. Accessibility is an objective and measurable concept relating to societal norms and legislation, and it is mainly measured on the population or group level.

When considering people with disabilities, some specific theories can be adopted to address mobility and walkability issues, such as the ecological model proposed by Lawton (1982), the congruence model of Kahana (1974), and the general adaptation theory (Carp and Carp, 1984). The first two theories primarily focused on the fit between the environment and either the individuals’ abilities or their needs. The third theory overcame such distinctions by incorporating both abilities and needs in a single model and by considering mobility as a key function in determining the degree of fit between the environment and the individual. The centrality of how an individual functions is, again, crucial to all the aforementioned theories and to determining a good person–environment fit. The studies that have utilized these theories have often aimed to identify how specific infrastructure elements (e.g., surface quality, sidewalk inclination, and light conditions) support walking behaviors.

### Environmental Psychology

Environmental psychology is an interdisciplinary field of study that strongly relies on psychological theories of human –environment transactions (Stokols, 1978; Canter and Craik, 1981; Bonnes and Secchiaroli, 1995; Gifford, 2014). The discipline is problem-oriented and linked to real-world situations that address the influence of the physical environment on people’s cognitive, emotional, and behavioral processes. Over time, there has also been an interest in regard to studies addressing environmental design for groups with disabilities (Tufvesson, 2007).

Like traffic planning, environmental psychology values the centrality of the individual. Crucial to each human – environment interaction are the individual psychosocial processes (e.g., perceived control and self-efficacy), which affect experience, behaviors, and overall wellbeing (Küller, 1991).

In the case of walking activities, the focus on underlying psychosocial processes is also acknowledged and believed to affect the quality of the experience itself. For example, using theories of restorative effects of nature, walking in natural settings has been shown to be beneficial for an individual’s emotional and cognitive regulation (Ottosson and Grahn, 2008; Adevi and Mårtensson, 2013). Self-regulation theories have also been employed, suggesting that walking is crucial for the personal development of a sense of belonging, attachment, and identification with place (Korpela, 1989; Metz, 2000). These latter feelings result from a correct balance between individual needs and environmental demands. Additionally, they are generally acknowledged to have a beneficial influence on quality of life outcomes (Burns, 1999; Lloyd and Auld, 2002).

Furthermore, the design of the physical environment and its perceived qualities have also been considered (Van Cauwenberg et al., 2012; Tribby et al., 2016). Such environmental qualities have been commonly defined as “*affordances*” for walking behaviors, i.e., properties of the setting perceived to be supportive for users’ needs and actions (Alfonzo, 2005; Cosco et al., 2010). The research field has long been concerned with people’s travel mode choices, drawing on theories of pro-environmental behavior to identify relevant antecedents (e.g., Richter et al., 2011). Nevertheless, the association between perceived environmental qualities and affective experience in the environment has recently been further developed in relation to walking behavior (Ferreira et al., 2016; Johansson et al., 2016). An affective experience refers to the individual attributes of emotional quality of a certain situation and to its related environmental characteristics (i.e., physical and social). Such an experience results in an emotional response that may, for example, encourage an individual to perceive an environment as positive and friendly because it supports the person’s needs and desires (Nasar, 2008).

Merging the perspectives of traffic planning and environmental psychology on walking allows for an integration of frameworks on human – environment transactions concerned about the psychological processes guiding human behavior with hands-on knowledge of planning, designing, and maintaining of the traffic environment for people with disabilities.

## Method

### The Process of Merging Environmental Psychology and Traffic Planning Into a Proposed Theoretical Model of Urban Walking Among People With Disabilities

Scientists experienced in working with mobility and vulnerable groups from traffic planning (*N* = 2) and environmental psychology (*N* = 2) discussed theories, concepts, and relevant themes relating to the topic of people with disabilities walking in residential neighborhoods in a series of five focus group meetings. Ethics approval for the focus groups was not required, as per institutional and national guidelines.

Each focus group meeting lasted approximately 2 h and was devoted to a specific topic. The meetings took place over months.

### Conceptual Analysis Process

The initial work performed was a conceptual analysis of urban walking among people with disabilities and addressed the connection between the environment and the individual during walking activities. Based upon knowledge from traffic planning and environmental psychology, several topics were considered: physical and social environments, urban walking definition, and individual abilities.

Early in the conceptual analysis process, the centrality of the individual characteristics, abilities, needs, and subjective experiences was discussed. As a result of the analysis, key concepts quickly surfaced among the teams (e.g., the importance of accounting for the subjective experiences of people with disabilities related to urban walking) (i.e., emotional response). The relevance of emotional responses related to the quality of the experience and the actual performance of urban walking is often overlooked from other theoretical models. However, it is fundamental for people with disabilities since their engagement with walking often resides in subjective constructs such as that of perceived self-efficacy (McAuley et al., 2007; Mullen et al., 2012).

Furthermore, the conceptual analysis identified the residential neighborhood as the most relevant and important setting for people with disabilities since it is the immediate spatial environment in which outdoor activities, such as that of urban walking, are performed (Dempster, 2008).

The next step in the conceptual analysis was to select a theoretical framework that could account for this holistic view of human–environment transactions in which physical, social, and bio-psychological aspects of walking could be accounted for and discussed in detail (Altman, 1976).

In parallel to these steps, key literature from traffic planning and environmental psychology were identified and shared between the scientists to support a discussion that could facilitate the merging of different knowledge within a single theoretical framework. The material was placed in a virtual library and utilized as a guide and a reference point during the workshops.

The Human Environment Interaction (HEI) model (Küller, 1991) was identified as a suitable framework for the purpose of merging the knowledge between the two disciplines since it provided the opportunity to address the centrality of an individual’s disabilities and subjective experience while accounting for the transaction with other environmental dimensions (i.e., physical and social environments) related to urban walking.

The HEI model is a holistic and user-centered framework used to analyze relationships between people and physical and social dimensions of specific settings.

According to this framework, each human – environment interaction is mediated by the individual’s affective experience. This suggests that a *basic emotional process* is the core of human – environment interplay. Depending on the quality of the interplay between the physical and social environments, the activity at hand, the individual’s characteristics and their prior experiences, different emotional experiences, and associated coping strategies are developed, which, in turn, will influence *wellbeing-related outcomes*.

The HEI model was originally developed in the field of environmental psychology, but, due to its intrinsic flexibility, it has been adopted as a framework in multidisciplinary studies addressing similar issues, settings, and target groups (Johansson, 2000, 2004, 2006; Marcheschi et al., 2014; Ferreira et al., 2016).

**FIGURE 1:**
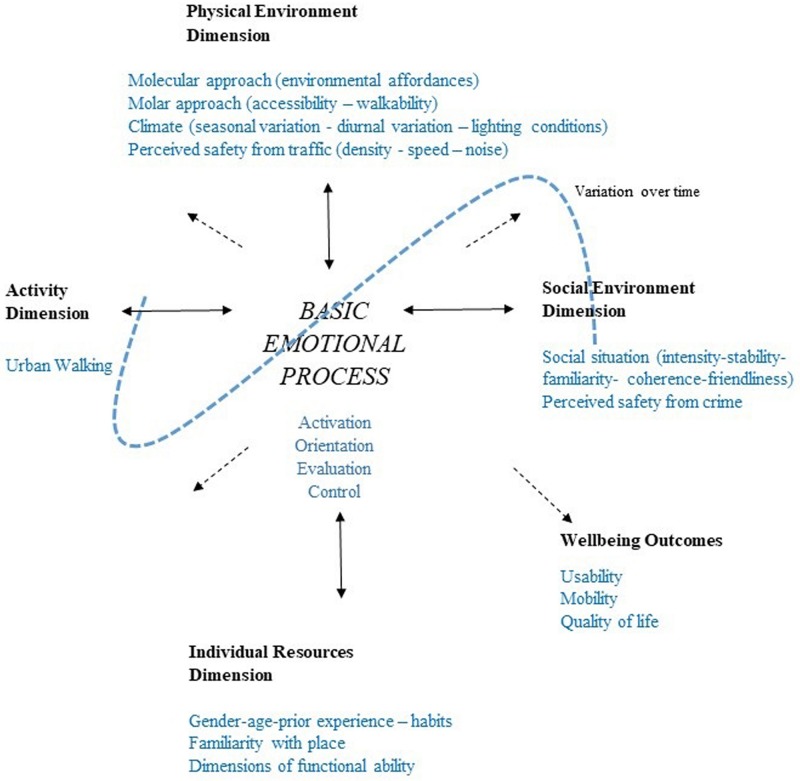
Operationalization of the HEI model for investigating urban walking among people with disabilities.

The framework of the HEI model is composed of four dimensions (*physical environment, social environment, activity, and individual resources*), one process (*basic emotional process*), and one outcome (*wellbeing*) (Figure 1).

An overarching description of the HEI model’s dimensions, process, and outcome was used as guide for the focus group discussions and the further operationalization of relevant concepts.

The *physical environment* is conceptualized as being composed of single features (molecular approach) and the result of how such features coexist in the overall environmental design (molar approach).

Two crucial aspects of the *social environment* seem to define the quality of the setting. *Crowding* refers to perceived human density and its related effects on the individual’s control over a certain situation, and *social support* refers to the perceived qualities held by social situations.

Human *activities* could be divided into three main categories: sleeping, everyday life, and extreme (dangerous). The model assumes that different activities have different demands on the other dimensions (physical, social, and individual), so it is important to accurately define the characteristics of the activity object of investigation.

The dimension describing *individual resources* relates to a person’s characteristics, such as age, gender, individual abilities, and prior experiences as well as their mental (cognitive and emotional) and behavioral strategies, which form the individual’s personal style. This is a coping scheme activated while interacting with the environment. This is particularly important to consider in relation to individuals with impaired abilities.

The *outcome* of the HEI model is the outcome of the *basic emotional processes*, conceptualized as the adjustment strategies activated by the individual to retain control over the situation. The degree to which such strategies proceed smoothly affects wellbeing-related outcomes.

## Results

The following section has summarized the results of the focus groups’ continued work by operationalizing the four dimensions, the basic emotional process, and the outcome of the HEI model for urban walking at a neighborhood level for people with disabilities.

An overview of the proposed version of the HEI model is shown in Figure 1.

### Activity Dimension

Walking at a neighborhood level was conceptualized in terms of a complex and dynamic interaction between physical and social characteristics of the setting as well as the bio-psychological aspects of the individual (i.e., physical abilities, perception, and experience). The definition of walking, proposed by Kärrholm et al. (2014), was adopted in this work, to support the communication across the disciplines and the operationalization of each discipline’s concept/construct of relevance.

Kärrholm et al. (2014) discussed “urban walking” as a dynamic and highly transformative activity – a heterogeneous process in which *where* and *how* to walk appeared to be reassessed by the individual throughout the experience (Kärrholm et al., 2014). From this perspective, walking behaviors are operationalized as series of different types of walking (assemblages) that are formed by the interaction with external (physical and social environment) and internal (psychological and personal characteristics) factors, which, in turn, shape the individual’s experience of walking. In this study, the notion of *urban walking* covered different levels of walking outcomes (physical, social, and bio-psychological), thus providing a description of the environment and it distinguishes between settings with different levels of environmental affordances for their users.

### Individual Resources Dimension

The dimension of individual resources concerns human functional abilities. This shows the varying degrees of individual abilities used to differentiate between different groups of people with disabilities and to define their needs. The concept of accessibility was used, stating that, on a group level, the grouping of the personal component must be homogeneous to enable assessment of the target environment (Iwarsson and Ståhl, 2003). Thus, a categorization used in the field of traffic planning into 12 dimensions of functional abilities, including mobility-related (*N* = 6), vision- and hearing-related (*N* = 3), cognitive-related (*N* = 2), and hypersensitivity-related (e.g., allergies) (*N* = 1) abilities [Almén and Ståhl, 2007 (CEN TC278/WG3, 2007)], was used. A summary of each dimension, the physical, corporeal and psychological areas involved, as well as the related difficulties connected with each condition, is provided in Table 1.

**TABLE 1:** Dimensions of functional ability (*N* = 12).

	**Dimension of functional ability**	**Description of functions and difficulties performing certain actions in the physical environment**
1	Wheelchair users, have function in arms and hands and in parts of torso and/or legs	Persons using manual wheelchairs generally manage to compensate for sudden movements caused by braking, vibrations, etc., and can cope with small, gradual changes of level, though they have difficulties opening heavy doors
2	Wheelchair users, partially reduced function in arms and hands and in parts of torso and/or legs, often having problems with balance	Persons generally using manual wheelchairs can have difficulties leaning sideways or forward without falling, have difficulties stretching to reach things, cannot use their bodies to compensate for sudden movements caused by braking, vibrations, etc., have difficulties with lateral slopes, have major difficulties opening heavy doors, generally lack a pincer grip, generally cannot cope with even very gradual changes of level, and can have problems with pain
3	Wheelchair users, reduced function in arms, torso and legs, have severe problems with balance	Persons generally using electric wheelchairs can have major difficulties leaning sideways or forward without falling, have limited ability to stretch to reach things, cannot use their bodies to compensate for sudden movements caused by braking, vibrations etc., have difficulties with lateral slopes, generally cannot cope with even very gradual changes of level, cannot open heavy doors, usually lack a pincer grip, and can have problems with pain
4	Reduced mobility, reduced function in legs and/or hips and/or spine, often have problems with balance	Persons generally using wheeled walkers, sticks, or crutches can have severe problems lifting their feet, difficulties walking backward or over uneven surfaces, can only walk short distances without rest, have difficulties with lateral slopes and long, gradual changes of level, can have problems stretching to reach things, cannot open heavy doors, and usually cannot manage even small, gradual changes of level
5	Reduced mobility, reduced function in arms and/or hands, limited reach – short in stature	Persons who have problems stretching to reach things, resisting a force, difficulties with heavy doors, etc., often experience great pain and often cannot carry bags
6	Reduced mobility, reduced strength and problems with balance	Persons with medical impairments, such as heart and lung disorders, can have major problems walking long distances and problems with rapid head movements
7	Visual impairment, can orientate using parts of sight, problems with balance	Persons who have great difficulty surveying their surroundings can have difficulties in perceiving changes in level/vertical changes, limited lateral or forward vision, reduced visual acuity, difficulty walking on uneven surfaces, need clear contrasts in light, have difficulties with lateral slopes, sometimes use a white cane and are helped by tactile contrasts, and have difficulty sorting important sounds in a noisy environment
8	Visual impairment, can orientate with white cane/guide dog, problems with balance	Persons who are blind or who have severely impaired vision can be unable to survey their surroundings, have difficulties in perceiving changes in level/vertical changes, have major problems walking on uneven surfaces, have major difficulties with lateral slopes, need clear tactile contrasts and/or clear indicators (also for guide dogs), and have difficulty sorting important sounds in a noisy environment
9	Hearing impairment, severe loss of hearing, or deafness	Persons who require hearing aids or are completely deaf can have difficulties surveying their surroundings, have great difficulties comprehending speech and sound, very distracted by background noise, have problems sorting or comprehending important sound information, and require clear visual information and, whenever applicable, an induction loop
10	Cognitive impairment, retardation	Persons with congenital impairment of central functions that cause problems in orientating, understanding layouts that are not logical, difficulties in managing sudden changes/making rapid evaluations/making assessments, and reading written text can usually understand pictogram images, but experience problems moving around due to the complex traffic environment
11	Cognitive impairment, acquired brain damage	Persons with a limited loss of some function that can lead to problems orientating and/or understanding layouts that are not logical, managing sudden changes, and reading written text can usually understand pictogram images, though can have problems moving around due to the complex traffic environment but can in some cases supplement using old knowledge
12	Allergy and hypersensitivity, allergic reactions and respiratory problems	Persons who suffer allergic reaction when exposed to scents, smoke, emissions, exhausts, pollen, and electricity can have problems being outdoors due to flowers with strong scents, wind-pollinated plants and/or dense traffic environment, often have problems walking long distances and/or have problems being in densely populated areas due to inhalation of allergens

*Source Almén and Ståhl, 2007 (CEN TC278/WG3, 2007).*

### Physical Environment Dimension

The physical environment, which in this specific case addresses a neighborhood unit of environmental analysis, was operationalized in terms of *molecular* and *molar* approaches (Küller, 1991).

*Molecular* approaches refer to the identification of infrastructure elements that are considered independent from one another. The challenge is to determine the degree to which certain elements are found in the environment. According to the nomenclature used in the field of environmental psychology, such elements can be defined in terms of *environmental affordances* since they support walking activities. Based on the dimensions of functional abilities and perceived difficulties in the physical environment presented in Table 1, the following affordances were selected for this proposed model: surface quality, crossing design, outdoor lighting, signs, environmental maintenance, resting possibilities, landscape characteristics, environmental variation, sounds, and odors.

*Molar* approaches, on the other hand, study the overall physical environment and are intended to show how well the affordances support users’ functioning and performance (i.e., an environmental quality index that captures the overarching person – environment fit).

In traffic planning, the concept that best describes such a molar view is *accessibility*. The focus is on the physical environment rather than the individual and, for this reason, it has been defined as an objective indicator of environmental quality (Iwarsson and Ståhl, 2003; Ståhl et al., 2010).

*Walkability* is a term that corresponds to accessibility and refers to how supportive a physical setting is for walking. Walkability can also be defined as an overarching indicator of environmental quality, addressing the friendliness of an area for walking activities (Kelly et al., 2007).

Ambience elements associated with climate conditions and perceived safety from traffic were also included and are considered critical for urban walking (e.g., Brown et al., 2007). Climate conditions comprise aspects relating to seasonal variation and perceived quality of light conditions, which are extremely important in terms of detection of obstacles and perceived quality of surfaces.

Perceived safety from traffic, on the other hand, refers to how the individual perceives traffic density, speed, noise, and how these interact with the person’s ability to cope with them (Brown et al., 2007; Wennberg et al., 2009).

### Social Environment Dimension

*Social environment* can be defined in terms of an overarching atmosphere or climate resulting from the perceived quality of the social interactions established in the setting (Moos et al., 1979; Wennberg et al., 2009). A positive social climate is very important for wellbeing outcomes among vulnerable groups in society, such as people with disabilities (Moos and Houts, 1968; Ittelson and Proshansky, 1970; Brunt and Hansson, 2002a, b; Brunt and Rask, 2007; Marcheschi et al., 2013).

Also, the presence of people moving around in the environment has a positive influence on the walking experience (Strath et al., 2007), but perceived crowding and a nuisance factor have negative influences on the walking experience (Brown et al., 2007). It is important to, in parallel, evaluate the perceived quality of the social situation and the physical environment to account for combined effects on urban walking among people with disabilities (Johansson et al., 2011; Mason et al., 2011).

The five aspects proposed by Küller (1991) to describe the quality of the social environment were chosen for this study – intensity, stability, familiarity, coherence, and friendliness. High levels of social intensity were discussed, in terms of stressors, that could contribute to an unbalanced emotional experience. This unbalanced emotional experience might, in turn, have a negative influence on urban walking for people with disabilities. On the other hand, the perception of a familiar and coherent social situation was considered supportive for a smooth development of the emotional processes associated with urban walking since it consequentially has a positive effect on the perception of quality when walking for people with disabilities (Janssen and Küller, 1989).

Aspects relating to perceived safety from crime were also considered as determinates of positive social climate outcomes (Brown et al., 2007). For this reason, information regarding perceived social incivility (e.g., vandalism, litter, and graffiti) as a source of nuisance from dangerous people and overall perceived safety were integrated in the model (Wennberg et al., 2009).

### Basic Emotional Process

The interaction between urban walking, individual resources (e.g., functional abilities of people with disabilities), and physical and social environments is mediated by the activation of the basic emotional process. This process serves to obtain information to evaluate the quality of the situation from the perspective of people with disabilities and for the purpose of performing urban walking. It therefore accounts for the affective responses associated with urban walking, and it proceeds in four phases: activation, orientation, evaluation, and control (Küller, 1991). Activation refers to the situation in which a certain stimulus (internal or external to the person) causes a temporary arousal in the individual’s emotional system. Such changes in the emotional state of activation are followed by an orientation and evaluation phase in which the individual’s attention is concentrated on identifying the stimulus and its quality. Depending on the degree to which the stimulus is perceived as positive or negative for the individual’s own interests, abilities, and needs (e.g., urban walking and perceived self-efficacy), different coping strategies will then be adopted (control phase).

Smooth development of the basic emotional process is associated with emotional balance, whereas all events or environmental features that hinder their development might result in emotional imbalance. Consequentially, the quality by which the basic emotional process develops impacts wellbeing outcomes among people with disabilities during urban walking.

### Wellbeing Outcome

Wellbeing outcomes are directly affected by the quality of the affective response (i.e., basic emotional process) experienced by people with disabilities during urban walking and the eventual need to develop adjustment strategies to cope with activities (e.g., stop or reduce walking).

Wellbeing and urban walking among people with disabilities are thereby strongly associated with the degree to which the person’s functioning and subjective experience fits with the environmental demands and supports the smooth development of the basic emotional processes and adjustment strategies (i.e., control over the situation) (HEI model). Prolonged needs for adjustments, due to a maladaptive person – environment fit, are known to negatively influence wellbeing outcomes (Küller, 1991).

In this study, wellbeing was operationalized in terms of the three concepts: *usability, mobility, and quality of life.* Usability is a subjective concept and is related to how an individual perceives the environment (i.e., the degree to which they can perform an activity), such as moving around in the environment (e.g., an urban neighborhood at a certain time). Usability varies due to individual or environmental conditions at that specific time (Iwarsson and Ståhl, 2003). A usable environment is one where an individual with impairments can function and perform their activities independently (World Health Organization [WHO], 2001).

Mobility is a broader concept that comprises several modes of travel, but, in this study, the focus was on pedestrian mobility (i.e., urban walking). The individual’s own perception of environmental usability is expected to influence the quality and frequency of mobility. Good levels of mobility and usability are known to support wellbeing outcomes by sustaining the individual’s sense of independence and purpose in life, but lower or decreased mobility is associated with powerlessness, depression, and isolation (Lawton and Nahemow, 1973; Banister and Bowling, 2004; Spinney et al., 2009). These latter feelings are often found in situations where disabilities are present and where the level of access to different opportunities is not equal (Casas, 2007).

In the scientific literature, quality of life is often used to operationalize wellbeing. Quality of life is a subjective evaluation of salient aspects of life comprised of physical and psychological health and quality of social and physical environmental factors (World Health Organization [WHO], 1993). In the context of urban walking, quality of life is suggested as an overall indicator of the individual’s satisfaction with life. For a person with disabilities, this is expected to increase when walking is performed in an environment that is experienced as usable. The concepts of usability and mobility are known to be crucial indicators of the quality of the walking experience, and, as expressed in quality of life, they are expected to affect wellbeing outcomes.

## Discussion

This work proposes an operationalized version of the Human Environment Interaction model (HEI model, Küller, 1991) as a tool that may facilitate an interdisciplinary analysis of urban walking among people with disabilities.

Previous models and theoretical frameworks developed within the field of traffic planning research have stressed the centrality of the individual’s needs, motivations, and active participation when it comes to the choice of engaging into more sustainable mode of transport, such as that of walking (Banister, 2008; Mateo-Babiano, 2016). However, the specific target group of people with disabilities appears to have been overlooked.

The model, proposed by this work, suggests that wellbeing outcomes (i.e., quality of life perception) in relation to urban walking among people with disabilities results from a dynamic interaction between health conditions (i.e., individual resources), environmental factors (i.e., physical and social environment), and the affective responses of humans. This operationalization is closely linked to the framework proposed by the International Classification of Functioning (ICF) in which wellbeing outcomes among people with disabilities result from the dynamic interplay between individual functioning (physical and psychological) and contextual factors (physical and social environments) (Ryan and Deci, 2001; World Health Organization [WHO], 2015).

The operationalized model was developed by systematically integrating knowledge and research findings from traffic planning and environmental psychology. The decision to choose the HEI model as a basis for the discussions in the focus groups proved very beneficial since it assisted the researchers to keep within the scope of walking at a neighborhood level for people with disabilities. However, accomplishing the goal of proposing a model is not without difficulties and poses many challenges to researchers involved in such a process. The proposed model suggests a multi-perspective strategy that includes the collection and compilation of information from different actors, in this case experts (environmental psychologists, architects, and traffic planners) and users (people with disabilities).

The model should, however, be regarded as a starting point rather than a final product. The flexibility of the model, explicated by the possibility to redefine the fundamentals of each dimension (i.e., individual resources, activity, and physical and social environments) as well as processes and outcomes, depending on the scope of the research and on the target population, allows for integration of further disciplines concerned with human – environment transactions (Johansson, 2006; Fyhri and Hjorthol, 2009). The aspect of flexibility is particularly relevant when investigating complex, dynamic, and heterogeneous human–environment interactions such as urban walking (Alfonzo, 2005; Kärrholm et al., 2014). Other disciplines that might benefit from further development of the proposed model are, for example, health sciences that may contribute by further elaborating on the individual’s capabilities, human geography that may deepen the meaning of place, and sociology could add the social context–power relations.

One of the major strengths of the proposed model is the focus on the subjective experiences people with disabilities have had of urban walking. This is expected to advance knowledge from previous theories, which have exclusively targeted the general population, by addressing diverse human factors and degrees of abilities involved in walking behaviors (Alfonzo, 2005).

The centrality of the individual is stressed by investigating how people with disabilities’ emotional processes (i.e., affective response) mediate the impact of the environment on urban walking. The subjective information generated makes it possible to identify associations between the characteristics of the environment and accessibility/usability, as well as, frequency and quality of mobility for people with disabilities and, in the long term, the related quality of life outcomes. Overall, this approach allows for focus on one single group as well as on multiple groups with disabilities at once, which hasn’t been addressed in previous models (Stafford and Baldwin, 2018).

Furthermore, with consideration for the influence that the physical environment design has on people with disabilities and their walking activities, the proposed model places great focus on the physical dimension of the environment by accounting for both molecular and molar features (Lawton and Nahemow, 1973; Imrie, 2003, 2012). The content of the physical environment dimension aroused particular interest in the discussions of the focus group meetings; most of the time was spent on this topic, which posed a particular challenge to the involved researchers. The discussions led to groupings that utilized the different traditions and theoretical approaches of the two disciplines. The proposed model clearly shows the necessity of including both molecular and molar aspects and a common terminology for these components was developed. From a planning perspective, the interaction between molecular and molar environmental levels is sometimes overlooked; however, for people with disabilities, the dynamic interplay between macro (e.g., proximity of services and street connectivity) and micro details (e.g., unevenness of the surface) have a significant influence on mobility possibilities (Clark et al., 2008). The proposed model allows empirical studies to produce relevant information about which physical elements of residential neighborhood areas are perceived to be more supportive for urban walking. Also, it allows conclusions to be drawn about how well the combined effects of those single elements support the walking needs of people with different disabilities.

A further strength of the proposed model is that the dimension of individual resource is related to, and described as, individual functional ability rather than disability. By this approach, the model highlights the theoretical model proposed by Lawton and Nahemow (1973) as well as the theoretical concept proposed by Iwarsson and Ståhl (2003) that the accessibility and usability of an environment are both relative concepts and a direct product of the relationship between the demand of the environment and the capacity of the individual. As such, it strengthens the understanding of the complexity in performing an activity such as walking.

A potential limitation of the proposed model is the requirement for knowledge and understanding about different disabilities and what consequences they might pose on an individual. The model is time consuming since the fundamentals of the dimensions must be carefully examined and operationalized by the combined work of different experts (i.e., traffic planning and environmental psychologists). However, the proposed model was developed through merging two disciplines, and that is how it is meant to be used (i.e., as a tool to facilitate the discourse and collaboration across different disciplines and social actors). In order to test the properties of the proposed model, the first step is to bring together the expertise of transdisciplinary teams around the topic of urban walking and people with disabilities. Practical information deriving from research projects (e.g., problem based and linked to real-life situations) is recommended. Specifically, a proposal is designed for pilot investigations in transdisciplinary contexts where collaborative approaches among relevant social actors (e.g., researchers and municipalities responsible for designing and maintaining of pedestrian environments) can be developed to support positive walking outcomes among people with disabilities.

The modified HEI model is thus seen as a tool that provides possibility to shift empirical research on walking from its main focus on the general population to the inclusion of diversity. This is to ensure opportunity to develop a new form of discourse in which social and spatial inclusion is considered for everyone. Through the model, we might be able to better understand the needs of different groups of people with disabilities during the activity of walking, and this improves our capability to create inclusive environmental design solutions (i.e., accessible and usable) that meet the needs of a wide spectrum of pedestrians (Iwarsson and Ståhl, 2003). This is in line with the principle of a Universal Design approach that seeks to support the needs of the maximum possible number of users but appears to be overlooked in regard to neighborhood planning and design (Lawton, 2001; Stafford and Baldwin, 2018). The empirical knowledge produced by the proposed model can feed into design recommendations (e.g., facilitators and obstacles of walking). This information can be used by policymakers and stakeholders in future planning of user-friendly neighborhood walking areas.

## Data Availability Statement

All datasets generated for this study are included in the article/supplementary material.

## Author Contributions

EM was the main writer of the text, and had the task of writing the overall manuscript and organize together the contribution and the merging of the two disciplines that are involved in this operationalization of the theoretical model. MJ had the initial idea of merging together the work of environmental psychology and traffic planning research, and further contribute by means of her expertise in environmental psychology with comments and feedback on the text. AS has together, with MJ, had the initial idea of merging together the work of these two disciplines. Moreover, AS has been actively involved in the writing of the section concerning the traffic planning perspective and have provided a great contribution in the structure of the manuscript. MA was an expert from the private sector that has supported AS in the discussion and formulation of the traffic planning section. The present paper was developed within the framework of EM Post Doctoral Studies at Lund University.

## Conflict of Interest

MA was employed by the company HinderfriDesign AB. The remaining authors declare that the research was conducted in the absence of any commercial or financial relationships that could be construed as a potential conflict of interest.
